# Motivation crowding effects on the intention for continued use of gamified fitness apps: a mixed-methods approach

**DOI:** 10.3389/fpsyg.2023.1286463

**Published:** 2024-01-10

**Authors:** Jialiang Huang, Jiang Chen, Liyun Zhou

**Affiliations:** ^1^School of Management, Guangzhou University, Guangzhou, China; ^2^College of Economics and Management, South China Agricultural University, Guangzhou, China

**Keywords:** intention for continued use, gamified fitness apps, gamification, motivation crowding theory, intrinsic motivations, extrinsic motivations

## Abstract

It has become an emerging idea for fitness apps to be gamified to intrinsically and extrinsically motivate user’s usage intention or behavior. For the long-term success of gamified fitness apps, understanding what and how various motivations affect continued use is critical. A combination of qualitative and quantitative methodologies was used to explore two research questions concerning gamified fitness applications. Specifically, the research questions focused on understanding the impacts of intrinsic and extrinsic motivations on continued usage. Additionally, the study aimed to investigate whether extrinsic motivations enhance or diminish the influence of intrinsic motivations. Results from qualitative study identified three intrinsic motivations (self-development, self-control and hedonic motivation) and two extrinsic motivations (social recognition and financial reward) in gamified fitness apps. Results from quantitative study indicated that intrinsic motivations (formed by self-development, self-control and hedonic motivation), financial reward and social recognition could significantly improve intention for continued use; and further, both financial reward and social recognition could crowd-in intrinsic motivations. This research offers insights into the phenomenon of motivation crowding effects on the intention to continue using gamified fitness apps.

## 1 Introduction

Fitness applications (fitness apps) include the mobile applications that promote or support users’ physical activity/exercise by monitoring or tracking physical activity, recording workout, prescribing exercise programs, offering exercise advices, or other ways (Barkley et al., 2020). With the rise of gamification, it has become an emerging idea for fitness apps to be gamified to enhance user’s psychological and behavioral outcomes (Lister et al., 2014). In this paper, gamified fitness apps (e.g., Nike+ apps, Huawei Health and Sport apps, Xiaomi Sport apps) pertains to fitness applications that incorporate diverse game design elements. These elements are integrated to amplify motivational affordances, thereby cultivating experiences akin to those encountered in games. Such elements encompass features like points, badges, leaderboards (PBL), team, competitive aspects, challenges, and even financial incentives. While the positive health benefits of gamified fitness apps are widely acknowledged (Whelan and Clohessy, 2020), gamification literatures have underlined the difficulty of achieving long-term success of gamified systems, as quite a few users discontinue their usage after their initial use of gamified systems (Liu et al., 2017; Suh et al., 2017). Literature has called for more attention to the success of gamified systems (Koivisto and Hamari, 2019; Yin et al., 2022), including a deep understanding of user’s continued use to gamified systems. Hence, understanding what and how various motivations drive continued use to gamified fitness apps could have theoretical implications for research and practical implications for fitness apps providers.

Gamified fitness apps have garnered attention from the academic community in recent years (Cheng, 2020; Whelan and Clohessy, 2020; Yang and Li, 2021; Li et al., 2023). Current literature primarily focuses on the impact of gamification on individuals’ fitness outcomes. Whelan and Clohessy (2020) demonstrate that social elements can enhance users’ passion for physical exercise. Yang and Li (2021) indicate that competition and interactivity may lead to privacy invasion and social overload, resulting in exhaustion. Li et al. (2023) discovered a U-shaped relationship between gamification competition and technological exhaustion. While these studies have confirmed the positive or negative effects of gamification on individuals’ fitness outcomes, the motivational factors for the continued use of gamified fitness apps have been seldom explored, even though such factors are essential for driving continued use in gamified apps (Wolf et al., 2018, 2020; Hassan et al., 2019). Specifically, our understanding of how various types of motivations interact to influence the continued use of gamified fitness apps has not yet been elucidated. To address these gaps, this paper investigates the continued use of gamified fitness apps from a motivational perspective, utilizing the Motivational Crowding Theory (MCT) as the research framework. The MCT sheds light on potential motivation crowding-in or crowding-out effects within gamified fitness apps.

From the lens of motivation theory, the use of gamified fitness apps can be attributed to a combination of intrinsic and extrinsic motivations (Hassan et al., 2019), where extrinsic motivations refer to “doing something due to a separable outcome (e.g., financial rewards, pressure and even verbal feedback),” and intrinsic motivations refer to “the doing of an activity for its inherent satisfactions” (Deci et al., 1999). For example, experience of enjoyment or flow induced by the gamified fitness apps can intrinsically drive use behavior; while desire for financial rewards for the completing tasks in the fitness apps can extrinsically drive use behavior. Gamification literatures also indicated that gamification could serve as both extrinsic motivations and intrinsic motivations for service use by providing a variety of rewards (Jang et al., 2018; Wolf et al., 2020). Hence, it’s arguably that gamified fitness apps could be driven by both intrinsic motivations and extrinsic motivations. Literature have also confirmed that user’s motivations for gamified apps use are multiple and can play crucial roles on continued use (Hamari and Koivisto, 2015; Hamari et al., 2018; James et al., 2019; Wolf et al., 2020).

The MCT has found extensive application across diverse contexts, facilitating an understanding of how extrinsic motivations can impact intrinsic motivations (Corduneanu et al., 2020; Graafland, 2020; Torre et al., 2020). The core of MCT lies the concept of the “motivation crowding effect,” which posits that extrinsic motivations have the potential to either diminish (crowd-out) or enhance (crowd-in) intrinsic motivations under distinct identifiable circumstances (Frey and Jegen, 2001). The motivation crowding effect is of great relevance for many IS contexts where various motivations take effects (Wu, 2019). As discussed above, this study argue that continued use of gamified fitness apps could be driven by both intrinsic and extrinsic motivations. Hence, this current study highlights the significance of investigating the potential motivation crowding effects in the gamified fitness apps. Nevertheless, seldom studies investigated whether and how different motivations can interact with each other in gamified fitness apps. To summarize, the objectives of this study are to investigate the following inquiries within the realm of gamified fitness apps:

RQ1: How do intrinsic and extrinsic motivations could drive continued use, respectively?

RQ2: Further, do extrinsic motivations crowd-in or crowd-out intrinsic motivations?

We used a mixed-methods design to address these questions. Initially, a qualitative investigation involving interviews was conducted to identify the motivations underpinning the utilization of gamified fitness apps. Results indicated the use of gamified fitness apps was driven concurrently by three intrinsic motivations (self-development, self-control and hedonic motivation) and two extrinsic motivations (social recognition and financial reward). Subsequently, a research model based on motivation crowding theory was proposed, followed by a quantitative study employing surveys to examine the research model. Our results indicate that both intrinsic motivations (formed by self-development, self-control and hedonic motivation) and extrinsic motivations (financial reward and social recognition) could increase intention for continued use; besides, these two types of extrinsic motivations could crowd-in (increase) intrinsic motivations. This study makes contributions to existing literature in multiple dimensions. To begin, our findings offer an enhanced comprehension of the motivations driving the use of gamified fitness apps, rooted in a user-centered viewpoint. Furthermore, we substantiate the direct impacts of intrinsic and extrinsic motivations on the intent to continued use of gamified fitness apps. Lastly, this study illuminates the intricate interplay between extrinsic and intrinsic motivations in shaping the intention to persist in using gamified fitness apps. This is accomplished by exploring the effects of motivation crowding and the mediating role of intrinsic motivations between extrinsic motivations and the intention to continue usage.

## 2 Literature review and theoretical foundations

### 2.1 Gamification

Typically, gamification entails the incorporation of game design components into contexts that are not inherently related to games (Deterding et al., 2011). In the domain of IS (Information Systems), Liu et al. (2017) provide a definition of gamification as the integration of game design elements into a designated system, while preserving its utilitarian functions. This stands in contrast to complete video games, which exist independently from real-world systems. Following these literatures, gamification in our research contexts refers to the incorporation of game design elements into the fitness apps while retaining its instrumental functions for promoting or supporting users’ physical activity/exercise. As an emerging design approach, gamification has attracted a large stream of researches in various contexts, including fitness/health/exercise (Cheng, 2020; Whelan and Clohessy, 2020; Yang and Li, 2021; Li et al., 2023), education (Sánchez Prieto et al., 2021; Behl et al., 2022; Chen and Liang, 2022; Ghai and Tandon, 2023; Oliveira et al., 2023; Zhang and Hasim, 2023), marketing (Zhang et al., 2021; Wu and Santana, 2022), sustainable consumption (Mulcahy et al., 2020), online community (Xi and Hamari, 2019).

Two main research questions have emerged from the gamification literature in IS domain, namely: (1) how gamification could affect individuals’ behavioral and psychological outcomes; and (2) why do people use or continued use gamified systems. To address the first question, studies have applied various theoretical perspectives, including Self-Determination Theory (SDT) (Morschheuser et al., 2019; Xi and Hamari, 2019), flow theory (Santhanam et al., 2016; Suh et al., 2017), perceived value (Mulcahy et al., 2020), psychological ownership (Xu et al., 2022), and motivational experience (Suh et al., 2017). As for the second question, research has offered several explanations, including utilitarian factors, hedonic factors, social factors, attitude (Hamari and Koivisto, 2015) and motivational experience (Hassan et al., 2019).

In general, compared with that on the first question, researches on the second topic are relatively less and insufficient (Koivisto and Hamari, 2019). Existing literatures suggest that motivations for continued use to gamified systems are diverse, but seldom studies have investigated whether and how different motivations could interact with each other in affecting continued use. Grounded in the framework of MCT, the primary objective of the present study is to enhance comprehension of this subject within the gamified fitness app context. This is achieved by delving into the dynamics of extrinsic motivations and intrinsic motivations, their influence on the intention to sustain usage, and additionally, examining whether extrinsic motivations have a crowd-in or crowd-out impact on intrinsic motivations.

### 2.2 Motivations for fitness apps use

Prior studies have investigated the factors driving use intention or behavior to fitness apps from various theories. Based on Technology Acceptance Model (TAM), Cho et al. (2015) find that ascertain that the perceived usefulness of fitness apps exhibits a positive correlation with the intention to use them. García-Fernández et al. (2020) take a stride in elucidating the impact of e-lifestyles on various associations with perceived ease of use, perceived usefulness, attitude, and intentions to use fitness apps. Draw from the Unified Theory of Acceptance and Use of Technology 2 (UTAUT2) model, Yuan et al. (2015) establish that factors such as hedonic motivations, price value, performance expectancy and habit collectively serve as predictors of users’ intentions to continue using fitness apps. Derived from the Expectation Confirmation Model (ECM), Li et al. (2019) ascertain that expectation confirmation serves as an internal driving force, while social comparison functions as an external driving force for the continued usage of fitness apps. Zhang and Xu (2020) reveal five psychological mechanism (confirmed usefulness, confirmed ease of use, satisfaction, fitness achievement and social connection) that determines college students’ continuance intention to use fitness apps. Taking the standpoint of social cognitive theory, Park et al. (2018) reveal that users’ self-efficacy, innovative propensity, outcome expectations, and engagement stand as pivotal factors influencing the intent to persist in using apps. Using the Uses and Gratifications (U&G) approach, Lee and Cho (2017) delve into how the satisfactions derived from utilizing diet and fitness apps can serve as motivations for users to sustain their engagement with these applications. Lewis et al. (2019) indicate that social interaction feature of fitness apps can offer virtual support for adults to improve engagement in physical activity.

While previous research has employed diverse theories to explore the motivations behind fitness app usage, and has highlighted the pivotal roles of both intrinsic and extrinsic motivations in driving such usage, these findings might not comprehensively elucidate the motivations underlying gamified fitness apps. This is due to the unique nature of gamification, which involves augmenting systems with motivational elements that foster experiences akin to those found in games (Huotari and Hamari, 2017). That is, the gamified fitness apps might trigger different motivations from those triggered by the non-gamified fitness apps. In addition, the motivation crowding effect has been seldom discussed in the (gamified) fitness apps context.

In contrast to studies that rely on conventional theories for Information Systems (IS) usage, the present study takes a user-centered approach. It aims to qualitatively uncover the underlying motivations driving the utilization of gamified fitness apps. Additionally, the study quantitatively examines how these motivations influence the intention to continue usage, while also investigating the presence of the motivation crowding effect within this specific context.

### 2.3 Motivation crowding theory

MCT suppose that extrinsic motivations have the potential to either diminish (crowd-out) or reinforce (crowd-in) intrinsic motivations under distinct and identifiable circumstances. When extrinsic motivations are perceived as controlling, they tend to lead to a crowding-out effect on intrinsic motivation; conversely, if these extrinsic motivations are perceived as supportive, a crowding-in effect on intrinsic motivation becomes apparent (Frey and Jegen, 2001). It’s important to highlight that while both intrinsic and extrinsic motivations can propel individual behavioral outcomes, it’s the intrinsic motivations that have the potential to enhance psychological well-being and the quality of effort exerted by individuals in a given task (Cerasoli et al., 2014). Hence, it’s of great significance to examine how extrinsic motivations can affect intrinsic motivations under the contexts where individuals’ behavior is driven by both.

In recent years, IS scholars have called for more efforts into the motivation crowding effects in various contexts (von Krogh et al., 2012), especially in gamified systems (Liu et al., 2017), as gamified systems can use various game design elements to intrinsically and extrinsically motivate user behavior. A few IS literature have confirmed the motivation crowding effects in different contexts, but the crowding in or out effects are inconsistent in various contexts. Osterloh and Rota (2007) demonstrate that extrinsic motivations can potentially displace voluntary sharing of software and knowledge, illustrating the occurrence of crowding-out effects. Hua et al. (2020) discover that monetary rewards and workplace spirituality can enhance the work engagement of Internet taxi drivers, thus supporting the occurrence of crowding-in effects within the realm of IT-enabled sharing platforms. Wu (2019) concludes that extrinsic motivations might either displace or amplify intrinsic motivation in various scenarios. Lombardi et al. (2020) suggest that knowledge-sharing rewards pose a significant obstacle to the favorable impact of both intrinsic motivation and lateral integrative mechanisms on knowledge sharing. Shen et al. (2022) draws on the MCT to explain the moderating effects of customer-initiated reviews and merchant-initiated replies on the relationship between negative online reviews and online sales performance. According to Sun et al. (2022), the effect of extrinsic motivations on knowledge contribution change from positive to negative as the level of prosocial motivation improves, supporting the crowding-out effect.

MCT has also been utilized to investigate how extrinsic rewards affect other individual economic behavior. For example, Wollbrant et al. (2022) find that efforts to enhance prosocial behavior through monetary rewards may have adverse effects. Likely, Ki (2022) indicate that the effect of monetary and promotion rewards on motivating government officials could be crowded out by public service motivation. Generally, these studies indicate that when utilizing external motivation to promote individual behavior, the crowding-out effect of motivation is present in various scenarios. In this study, it is postulated that the context of gamified fitness apps could potentially exhibit motivation crowding effects. However, there has been limited exploration of motivation crowding effects within the domain of gamification studies as far as current knowledge suggests.

In addition, we believe that fitness literatures that investigate the effects of extrinsic motivation may provide some insights into the motivation crowding effects in the gamified fitness apps. For example, Charness and Gneezy (2009) find that there is potential for financial incentive in the development of good health habits. In the context of fitness apps, Plangger et al. (2019) find that tangible extrinsic rewards can improve users’ activity levels in the gamified health apps. Oyibo and Vassileva (2019) find that rewards can predict competitive behavior in fitness apps. Whelan and Clohessy (2020) show that extrinsic social rewards (social norms, recognition and reciprocal benefits) can have enhance users’ passion in the fitness apps.

To sum up, while the literature highlights the coexistence of both extrinsic and intrinsic motivations within gamified fitness apps, limited attention has been paid to the potential motivation crowding effects in the gamified fitness apps. Relevant studies primarily concentrate on exploring the influence of extrinsic motivations, such as tangible rewards and social rewards, on the transformation of fitness-related behaviors and the motivations underlying the utilization of fitness apps. The current study aims to provide insights into this issue by investigating the motivation crowding effects for the continued intention to use of gamified fitness apps.

## 3 Research design

This study employs a mixed-methods approach, which combines both quantitative and qualitative research methods. This approach was chosen to provide a more comprehensive and nuanced understanding of the research phenomena, enhancing the reliability of the results (Miles and Huberman, 1984; French et al., 2017; Hua et al., 2020). Integrating both qualitative and quantitative methods offers mutual reinforcement (Venkatesh et al., 2013). Qualitative methods are well-suited for addressing explanatory research, enabling in-depth insights into a phenomenon and the generation of novel theoretical insights. In contrast, quantitative methods are effective for conducting confirmatory studies that involve testing theories and relationships (Zhang, 2017; Hua et al., 2020). This mixed-methods design confers several advantages compared to relying on a single method of research. It allows for the simultaneous exploration of both confirmatory and exploratory research questions, providing a well-rounded approach to data collection and analysis (Venkatesh et al., 2016).

As discussed in the review section, even though studies have applied various theories to investigated the factors for fitness apps use, limited attention has been paid to the motivations for gamified fitness apps use, especially from a user-centered perspective. Gamification can enhance the motivational affordances of the system (Koivisto and Hamari, 2019), and thus change the users’ motivations for systems use. Consequently, this study undertakes a qualitative study to uncover the motivations behind the usage of gamified fitness apps, thereby enriching the comprehension of the factors driving their adoption and enhancing the reliability of findings. Furthermore, the investigation into motivation crowding effects concerning the ongoing intention to use gamified fitness apps remains unexplored. When considering these factors collectively, this study deals with both exploratory and confirmatory research inquiries. As a result, a mixed-methods design is deemed suitable and appropriate for this study, given its capability to accommodate the diverse aspects of the research objectives.

**Figure 1:**
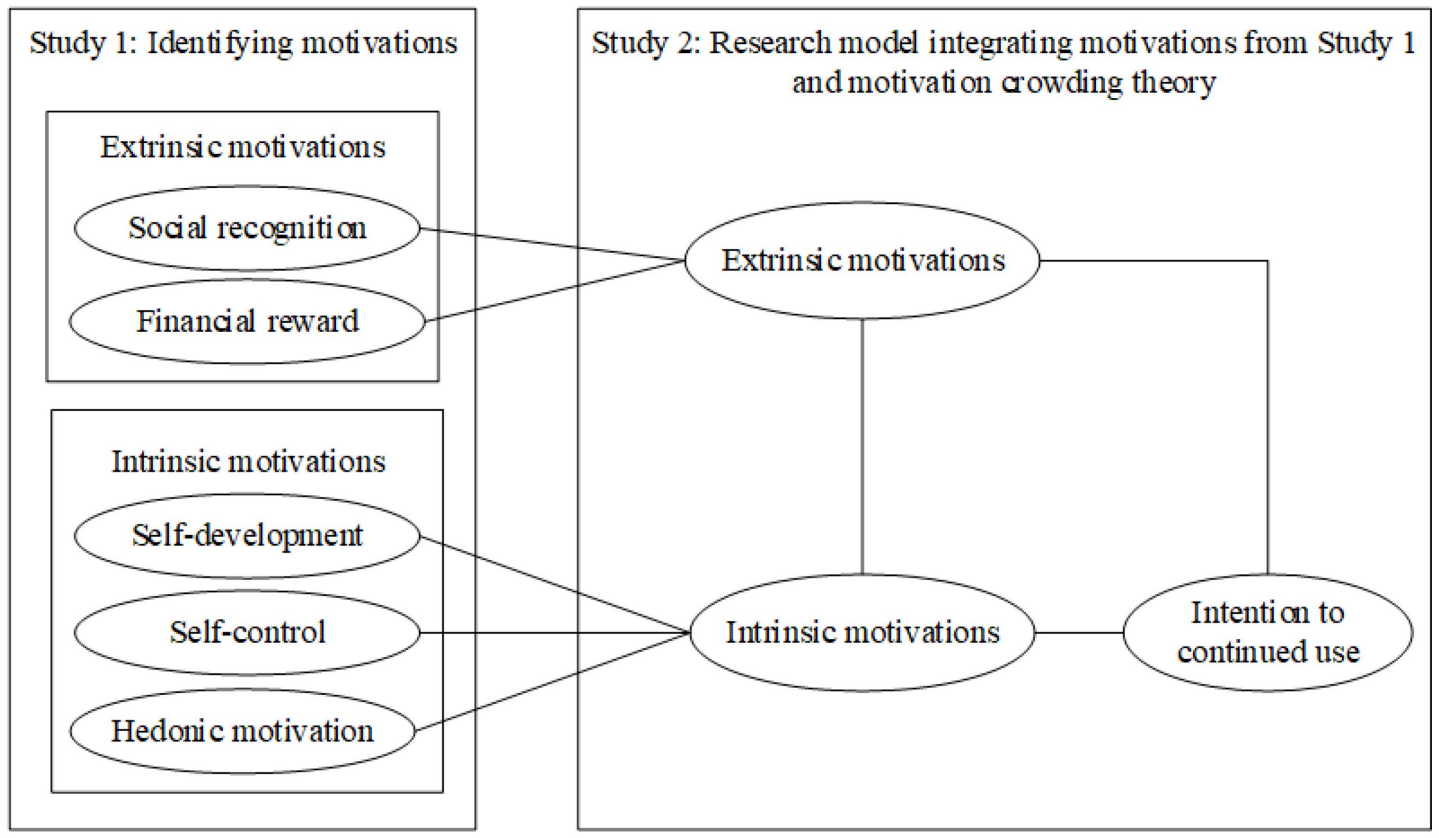
Relationship between two studies.

To be specific, this study employed a two-phased approach, consisting of a qualitative study (Study 1) conducted through interviews and a subsequent quantitative study (Study 2) carried out via a survey methodology (French et al., 2017; Zhang, 2017; Hua et al., 2020). The interrelationship between these two studies is visually depicted in Figure 1. During Study 1, a qualitative investigation involving 30 semi-structured interviews was undertaken to identify five motivations driving the utilization of gamified fitness apps. Subsequently, these motivations were categorized into intrinsic and extrinsic categories based on their conceptual nature, drawing upon the work of Deci et al. (1999). In Study 2, a research model was formulated. This model was built upon the principles of the motivation crowding theory, integrating insights gleaned from Study 1. An online survey was administered to test its validity and applicability.

## 4 Study 1: Qualitative research—interviews

### 4.1 Data collection

The sample contained actual users of gamified fitness apps in China to ensure external validity (Wolf et al., 2020). We searched the Huawei App Store and the Xiaomi App Store (two of the largest app stores in China) for the actual gamified fitness apps. Finally, we determine “Keep (App),” “Huawei Sports and Health (App)” and “Xiaomi Sports (App)” as the research contexts, based on two criteria. Firstly, the apps’ cumulative downloads from the two App stores surpassed 100 million, signifying their status as three of the most widely used fitness apps in China. Secondly, these selected apps were notable for incorporating an extensive array of game design elements, thereby ensuring a substantial degree of gamification to motivate user behavior. Upon closer examination, it was evident that all three apps shared a similar suite of game design elements. These elements, constituting the gamification features of the apps, are described in Table 1.

**Table 1:** Brief description of the game design elements in the selected fitness apps.

Elements	Description	Source
Badges	Visual representations of achievements, be collected within the gamification environment	Werbach and Hunter (2012)
Levels	A system of advancing in the gamified apps by collecting a certain amount of points or carrying out specific actions	Gatautis et al. (2016)
Social network features	Messages, blogs, chat, sharing and connection to social networks	Xi and Hamari (2019)
Points	Points are rewarded for the successful accomplishment of specified activities within the gamified apps, and they allow the users’ fitness behavior to be measured	Werbach and Hunter (2012) and Sailer et al. (2017)
Progress bars	Performance graphs provide information about users’ performance compared to their preceding performance during a task	Sailer et al. (2017)
Quests	Quests are little tasks that users have to fulfill within a fitness program	Xi and Hamari (2019)
Challenge	Challenge represents the user’s desire to overcome the challenge, which corresponds to the presence of “hints” provided by the gamified apps	Mulcahy et al. (2020)
Team	Defined groups of players that work together toward a shared objective	Sailer et al. (2017)
Leaderboards	Leaderboards rank users according to their relative success, measuring them against a certain success criterion	Sailer et al. (2017)
User profiles	User profiles allow users to check their personal fitness data, e.g., amount of physical activity and weight	Wolf et al. (2020)
Financial reward	Points can be redeemed for financial reward such as cash or gift	Koivisto and Hamari (2019)

To invite participants for the qualitative study, we used a convenient sample and networking by contacting users of the three selected gamified fitness apps that the researchers are familiar with, following French et al. (2017). We asked all participations to show the interfaces of the targeted gamified fitness apps that installed on their mobile phone and their created accounts, to confirm that they are actual users of the targeted gamified fitness apps. Then, multiple choice questions related to the game design elements were used to confirm that the participants have interacted with the game design elements within the apps.

Motivations for using gamified fitness apps were identified through interviews with self-reported participants. Following Hua et al. (2020), a two-stage approach was used to collect qualitative data: (1) 2 pilot interviews and (2) 30 semi-structured interviews. To ensure the clarity and comprehensibility of the interview questions, an initial phase of 2 pilot interviews was conducted. The feedback obtained from these pilot interviews corroborated the suitability of the interview questions. Subsequently, a total of 30 participants were invited to take part in semi-structured interviews aimed at gathering qualitative data pertaining to motivations driving the usage of gamified fitness apps. The characteristics of the participants are presented in Table 2.

**Table 2:** Basic characteristics of the participants for semi-structured interviews.

		Frequency
Gender	Male	13
	Female	17
Age	<20	3
	20–30	15
	>30	12
Using experience	< 6 months	1
	6 months–1 year	7
	1–2 years	17
	>2 years	5
Number of selected elements	1–3	3
	4–6	10
	7–11	17
Length for interview	5–10 min	4
	11–15 min	16
	16–20 min	10

Two researchers conducted the semi-structured interviews by the following steps. First, the participants were informed that the purpose of the interview was to explore why they use the fitness apps, and that their responses were only for academic research and kept anonymous. Second, participants were asked to provide the basic information including demographics, gamified fitness apps in used, interaction with game design element, and their using experience and duration. Third, participants were asked about their motivations for using gamified fitness apps by three standard questions, i.e., “Why do you use the (name of the gamified fitness app in use)?,” “What are the major benefits of using this app?,” and “please comment on the experience of using this app”; besides, the researchers might ask additional questions depending on the responses to each standard question, which was in line with a semi-structured interview method and might provide greater depth in each interview (Zhang, 2017). All the interviews were taped and then transcribed. Besides, participants were asked to write down their answers to better ensure accuracy of responses (French et al., 2017). The original interviews were performed in Chinese. Every interview lasted 5–20 min.

### 4.2 Data coding

Two coders performed the qualitative data coding following steps described by prior studies (French et al., 2017; Zhang, 2017). Frist, the coders conducted open coding and extracted the first-order concepts, i.e., the statements from the original semi-structural interviews script that is relevant to understand the motivations for using gamified fitness apps (Wu, 2019). Two examples of the first-order concepts are “This app provides rich professional running tutorials” and “I can follow the app for training.” Second, the first-order concepts were compared and grouped the similar first-order concepts (expressed differently) into the second-order themes, i.e., the labels the first-order concepts that describe the same motivations. For instance, the two first-order concepts above both describe the motivation for gaining knowledge, and hence could be grouped into a second-order theme as “knowledge.” Third, the coders compared the second-order themes and aggregated the similar second-order themes into higher-order theoretical constructs if possible. For example, the two second-order themes, “knowledge” and “image” aggregated to a higher-level construct as “self-development.”

After the coding was completed, the interrater reliability was used to evaluate the reliability of the coding; the two coders compared their coding to identify instances to identify the instances where they matched (French et al., 2017). The Cohen’s Kappa value was 0.71. After that, the coders further discussed instances that did not match and decided whether they could reach an agreement, and only those instances that were agreed on a coding are retained (French et al., 2017).

### 4.3 Results

After the evaluation, a total of ten motivations were identified from the data coding. Following Hua et al. (2020) and French et al. (2017), only the motivations that frequently mentioned (3 or more times) were selected for further analysis. As a result, Study 1 identified five motivations (shown in Table 3) as the main constructs in Study 2.

**Table 3:** Motivations for using gamified fitness apps.

Motivation	Times mentioned	# of interviewees	Motivation	Times mentioned	# of interviewees
Self-development	33	23	Social recognition	13	9
Self-control	26	17	Financial reward	8	5
Hedonic motivation	17	11			

Self-development, the most frequently mentioned motivation (33 times and 23 interviewees), refers to a persistent pursuit of personal well-being enhancement, achieved through processes such as learning from peers within the domain, seeking feedback, and honing one’s abilities and competencies (Bauer and McAdams, 2004; Oreg and Nov, 2008; Wolf et al., 2020). In gamified fitness apps, users could perceive self-development when they interact with certain game design elements such as challenge, informational feedback and user profiles. For example, users could feel their progress with their fitness data record and feedback provided by the apps. A coded instance is “*I have used this app for several months, and I can record my exercise data in this app. I can feel that my figure is getting much better, which made me felt a sense of achievement*.” Also, some users could have experiences for self-development when they overcome challenges, for instance “*This app can provide me with challenging task according to my personal situation*” In addition, users can get professional guidance or knowledge related to fitness from the apps, and feel an increase of their abilities and skills, for instance “*I have a habit of running. This app provides rich professional running tutorials, which helps me learn a lot knowledge and guides me to run more efficiently*.”

Self-control, the second frequently mentioned motivation (26 times, 17 interviewees), encompasses the ability to modify or regulate one’s emotional and behavioral reactions to facilitate the achievement of long-term objectives (Muraven and Baumeister, 2000; Baumeister et al., 2007; Jeong et al., 2016). Game design elements such as training task, reminder and feedback in gamified fitness apps can help users to stick to a long-term exercise plan. Some users expressed that the gamified fitness apps could help them keep exercising for a long-term, such as “*I kept exercised every day for a month since I used this app*” and “*This app would remind me to keep on exercise when I sometimes forget to do it*.” This motivation is stronger for users who want to keep fit but without strong self-control, for instance “*I hope to get in shape, especially in the summer, but I am not a very self-controlled person. I can follow the training video to form an exercise habit*.”

The third frequently mentioned motivation is hedonic motivation (17 times, 11 interviewees). Hedonic motivation is characterized as the enjoyment or pleasure derived from utilizing a system (Venkatesh et al., 2012), Typical words for this motivation include “enjoy,” “interesting” and “fun.” It’s not surprising that hedonic motivation is one main reason for using gamified fitness apps, as it’s a main factor for using gamified services (Hamari and Koivisto, 2015). Thus, game design elements in the fitness apps can induce gameful experience for fitness activities. Besides, the immersion-related elements such as music and video can create a sense of fun or enjoyment for users. A few coded instances related to hedonic motivations are “*It’s a very interesting and creative app. I enjoy using this app*” and “*Listening to the music by this app while doing exercise is more interesting*.”

The four motivation is social recognition (13times, 9 interviewees) which refers to the desire to gain social feedback (e.g., praise) on their performance from others (Hsu and Lin, 2008). Many fitness apps today use social-related elements to provide user with a sense of recognition, acknowledgment, or appreciation. Some users express a desire for getting approval from others in the form of virtual praise. For example, a user said that “*I can get ‘likes’ if I get a high ranking in the list*.” In addition, elements such as leader boards and data sharing can induce an experience of social comparison which is related to social recognition (Wolf et al., 2020). Users might be motivated to use gamified fitness apps and share their fitness data when they see others doing the same, for instance “*Lots of friends will share their exercise performance in WeChat, and I will do the same*.”

The last motivation is financial reward (8 times, 5 interviewees). Financial reward is a common kind of external intervention to motivate individual behavior (Hua et al., 2020). Financial reward is a common mean to extrinsically motivate users to engage in challenges or task activities in gamified fitness apps. Many gamified fitness apps allow users to exchange their points for coupons or even cash. Some coded instances for expressing motivation for financial reward are “*I like earning points because they can be redeemed for coupon*s” and “*It’s a good app because the lucky draws can greatly improve my enthusiasm for exercises*.”

Following the identification of motivations, a subsequent classification was conducted, categorizing them into either intrinsic motivations or extrinsic motivations. On the one hand, intrinsic motivations refer to “the doing of an activity for its inherent satisfactions.” Intrinsic motivations are often rooted in the fulfillment of three fundamental psychological needs: competence, autonomy, and social relatedness (Deci and Ryan, 2000). Among those five motivations, self-development, self-control, and hedonic motivation were classified as intrinsic motivations in gamified fitness apps. Self-development is about overcoming challenges and hence relates to the need for competence. Self-control involves taking control of one’s emotional and behavioral responses, which is related to need for autonomy. Hedonic motivation involves the fun or enjoyment derived from using the service itself, which should be a kind of intrinsic motivation.

On the other hand, extrinsic motivations refer to “doing something due to a separable outcome (e.g., monetary rewards, pressure and even verbal feedback)” (Deci et al., 1999). Individuals who are extrinsically motivated focus more on the outcomes rather than the process of an activity (Kivetz, 2004). External rewards, such as monetary compensation or social status, are provided in return for engaging in specific behaviors or activities (Zichermann and Linder, 2010). In the gamified fitness apps, users could be extrinsically motivated via social recognition and accumulation of material (financial) rewards (Jang et al., 2018). In line with these arguments, social recognition and financial reward were classified as extrinsic motivations in this study. In summary, Study 1 provided three intrinsic motivations (i.e., self-development, self-control and hedonic motivation) and two extrinsic motivations (social recognition and financial reward) for using gamified fitness apps.

## 5 Study 2: quantitative research—survey

Building upon the theoretical framework of the MCT and drawing insights from the outcomes of Study 1, Study 2 undertook a quantitative investigation through a survey. This study aimed to examine the impacts of intrinsic and extrinsic motivations on continued use, as well as the motivation crowding effects.

### 5.1 Research model and hypotheses

**Figure 2:**
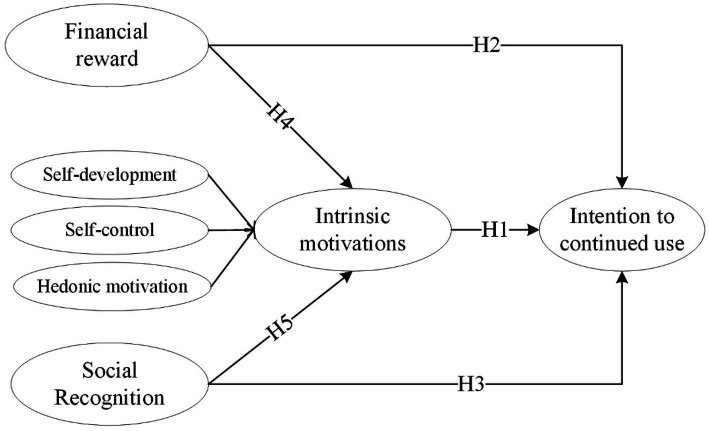
Research model in Study 2.

Figure 2 illustrated the research model. As mentioned in study 1, this study considered two types of extrinsic motivations, namely social recognition and financial reward. Besides, following Hua et al. (2020), this study treated intrinsic motivations as a second-order formative construct that consists of three dimensions of intrinsic motivations identified from Study 1 for two reasons. First, one of our main interests focuses on the motivation crowding effect, wherein extrinsic motivations impact intrinsic motivations at a more elevated conceptual level. In this situation, the adoption of second-order constructs holds the potential for enhanced theoretical conciseness, mitigation of model intricacy, and alignment of abstraction levels between predictive and outcome variables in research frameworks models (Becker et al., 2012). Second, formative representation is preferred over reflective because those three first order intrinsic motivations are distinct in nature and not interchangeable.

#### 5.1.1 Intrinsic motivations and intention for continued use

Psychological researches consider intrinsic motivations as one of the strongest forces behind individuals’ behavior outcomes (Deci and Ryan, 2000; Xi and Hamari, 2019). Intrinsic motivations exhibit a positive correlation with both the magnitude and caliber of effort exerted by individuals in a given task (Cerasoli et al., 2014). Users are inherently driven to interact with the gamified system solely for the intrinsic pleasure of engagement (Koivisto and Hamari, 2019). Gamification holds the potential to augment services by incorporating features that facilitate game-like experiences to enhance user’s overall value creation (Huotari and Hamari, 2012). Intrinsic motivations such as enjoyment can play crucial roles in continued use or loyalty in gamification contexts (Hwang and Choi, 2020; Mulcahy et al., 2020; Wolf et al., 2020). Gamified fitness apps can intrinsically motivate users’ usage behavior via implementing various game design elements; and it is likely that users would continue to use gamified service when they are intrinsically motivated. Hence, we propose:

*H1*: Intrinsic motivations positively affect intention for continued use of gamified fitness apps.

#### 5.1.2 Financial reward and intention for continued use

Financial reward (e.g., monetary reward, gifts, lucky draw) is widely used in the online service including gamified fitness apps to engage user, because the idea of using financial reward to drive individuals’ behavior is grounded in economic and psychological literature (Sun et al., 2017). Financial reward is also an implemented affordance in gamified system (Koivisto and Hamari, 2019). In addition, when financial reward is introduced, users may see their engagement (e.g., time and effort) as a “social exchange process” (Emerson, 1976), and cost–benefit analysis might occur; users would perceive their engagement more valuable when they receive financial reward. Users driven by extrinsic motivations are predisposed to engage more actively with the gamification elements integrated within fitness applications (James et al., 2019). Therefore, apart from intrinsic motivations, users may also find extrinsic motivation through financial reward to continuously use gamified fitness apps. Hence, we propose:

*H2*: Financial reward positively affects intention for continued use of gamified fitness apps.

#### 5.1.3 Social recognition and intention for continued use

It’s widely admitted in psychological research that individuals pursue social recognition from others (Hwang and Francesco, 2004), and receiving recognition from a highly esteemed reference group significantly contributes to psychological well-being (Wolf et al., 2020). Social recognition, functioning as a form of extrinsic reward, possesses the potential to exert a robust and favorable influence on the intentions to sustain the use of ICT (Hernandez et al., 2011). When a service offers social recognition to users, their perception would be more positive, and hence enhance their attitude as well as continued use (Hamari and Koivisto, 2015). Stragier et al. (2016) indicate that social motivations could predict exercise app usage. It’s popular for fitness apps to enable their users to provide social feedback to others, because peer recognition has a strong impact on users’ passion (Whelan and Clohessy, 2020). Gamified fitness apps can satisfy user’s desire for social recognition via social elements such as community and leaderboard, which can extrinsically drive continued use. Hence, we propose:

*H3*: Social recognition positively affects intention for continued use of gamified fitness apps.

#### 5.1.4 Crowding-in effects of financial reward and social recognition

According to MCT, extrinsic motivations, including monetary or non-monetary, might crowd-in (increase) or crowd-out (decrease) intrinsic motivations; whether extrinsic motivations crowd-in or crowd-out intrinsic motivations depends on how individuals perceived them as controlling or supportive (Frey and Jegen, 2001).

Extrinsic motivations have the potential to be internalized and transformed into intrinsic motivations when there are ambient supports for the needs for autonomy, competence and relatedness (Deci and Ryan, 2000). Thus, if users can perceive the gratification of these psychological needs when they attain extrinsic rewards, they would develop intrinsic motivation to participate actively in gamified services (Xi and Hamari, 2019). Financial incentive is likely to viable approach that can effectively bolster intrinsic motivations without sacrificing individual autonomy (Hua et al., 2020). In the contexts of gamified fitness apps, financial reward is commonly used as a reward for completing the challenges or tasks, and voluntary rather than mandatory; hence, it should not damage users’ needs for autonomy and even satisfy the needs for competence. In addition, financial reward includes the form of the lucky draw which could trigger users’ enjoyment or excitement because of its uncertainty (Goldsmith and Amir, 2010). According to the above analysis, we propose:

*H4*: Financial reward is positively associated with intrinsic motivations in gamified fitness apps.

On the other hand, social recognition as a source of extrinsic motivations might also crowd-in intrinsic motivations if it supports the fulfillment of the basic psychological needs. Social recognition is commonly regarded as a positive experiential phenomenon (Whelan and Clohessy, 2020). Upon receiving social recognition from fellow users, individuals may experience a heightened sense of connection to their peers (Shiau et al., 2018). Experiencing social connectedness is directly associated with the need satisfaction of social relatedness (Deci and Ryan, 2000). Besides, literatures also indicate that social feedback can improve users’ informed decision making (Hassan et al., 2019), and thus users might regard it as supportive rather than controlling. Game design components like “likes” or leaderboards can facilitate users in obtaining social feedback and engaging in social comparison. These encounters have a positive correlation with intrinsic satisfaction, as individuals often construct their self-concept based on social feedback (Wolf et al., 2020). Simultaneously, social competition can cultivate a sense of belonging (van Roy and Zaman, 2019). According to the above analysis, we propose:

*H5*: Social recognition positively affects intrinsic motivations in gamified fitness apps.

### 5.2 Measurement

The present study customized items from existing literature to align with the research context. For three types of intrinsic motivations, we used items from Wolf et al. (2020) to access self-development, items from Tangney et al. (2004) to access self-control and items from Venkatesh et al. (2012) to evaluate hedonic motivation. Items pertaining to financial reward were derived from Hua et al. (2020), while those associated with social recognition were adapted from Hamari and Koivisto (2015). Intention to continue use was evaluated using items from Venkatesh et al. (2012). To measure these constructs, a seven-point Likert scale was employed, spanning from 1 (strongly disagree) to 7 (strongly agree). The complete array of items can be found in Table 4.

**Table 4:** Items for construct.

Construct	Items	Source
Self-development (SD)	SD1: The app helps me to reach my objectives	Wolf et al. (2020)
	SD2: The app helps me to face a challenging task	
	SD3: The app helps me to develop myself	
Self-control (SC)	SC1: I become less lazy with this app	Tangney et al. (2004)
	SC2: I can form exercise habits with this app	
	SC3: I am more self-discipline with this app	
	SC4: I am able to exercise effectively toward long-term goals with this app	
Hedonic motivation (HM)	HM1: Using the app is fun	Venkatesh et al. (2012)
	HM2: Using the app is enjoyable	
	HM3: Using the app is very entertaining	
Financial reward (FR)	FR1: The coupons provided by the app inspires me	Hua et al. (2020)
	FR2: The lucky draw provided by the app inspire me	
	FR3: I am happy if I get financial reward after complete the challenges in the app	
Social recognition (SR)	SR1: I feel good when my achievements in the app are noticed	Hamari and Koivisto (2015)
	SR2: I like it when my friends comment and like my usage in the app	
	SR3: I like it when my friends notice my achievements in the app	
	SR4: It feels good to notice that my friends have browsed my achievement in the app	
Intention for continued use (CS)	CS1: I intend to continue using this app in the future	Venkatesh et al. (2012)
	CS2: I will always try to use this app when I do exercise	
	CS3: It is likely that I will use the app more often rather than less often during the next couple months	

### 5.3 Pilot study and formal study design

A pilot study (*n* = 35) was performed to check the measurement quality. Feedbacks from respondents indicated that the items used in this study were well understood and of good content validity. Besides, we employed Cronbach’s coefficient alpha to assess the reliability of the constructs. Results showed that each construct had a sound construct reliability (>0.7).

Primary data for the formal study were gathered via an online survey conducted in China. Subjects were users of gamified fitness apps in consistent with Study 1. We engaged the services of www.wjx.cn, a reputable online survey firm equipped with a vast user database comprising millions of individuals across China (Xu et al., 2017). This platform offers specialized sample services, enabling researchers to disseminate surveys to specific target groups that align with predetermined criteria such as age, gender, and other contextual variables.

Our survey included several sections. First, respondents were shown with the survey instructions including the research purpose, context and data use statements. Second, a screening question was used to make sure that respondents were users of the targeted gamified fitness apps. Third, respondents were asked to complete the were requested to diligently respond to the content of the survey. Four, respondents were invited to provide responses to inquiries regarding demographic information. At last, respondents were extended gratitude for their active participation in the survey and were offered the opportunity to enter a lucky draw.

Initially, we received 700 questionnaires. Subsequently, we undertook the removal of 186 invalidated questionnaires, which exhibited uniform or nearly identical responses across most questions, demonstrated logical inconsistencies, or displayed completion times of less than 1 min. a cumulative sum of 514 reliable responses was acquired and subsequently utilized to assess the viability of the research model. Table 5 presents the demographic characteristics and experience of the respondents. 206 (308) respondents are male (female). As for age, about 15% are aged below 20, about 37% are aged between 20–30, about 30% are between 31–40, and about 18% are above 40. Regarding education, most respondents have college or higher degree. About 76% of the respondents have experienced longer than 6 months.

**Table 5:** Demographic characteristics and experience of the respondents.

		Frequency	Percentage
Gender	Male	206	40%
	Female	308	60%
Age	<20	78	15%
	20–30	192	37%
	31–40	152	30%
	>40	92	18%
Education	High school or below	55	11%
	College	156	30%
	Bachelor	188	37%
	Graduate	115	22%
Length of experience	<6 months	123	24%
	6 months–1 year	139	27%
	1–2 years	165	32%
	>2 years	87	17%

### 5.4 Data analysis and results

We employed the component-based Partial Least Squares Structural Equation Modeling (PLS-SEM) technique, utilizing Smart PLS 3.2.8 software, to scrutinize both the measurement and research models. Our research framework incorporates a second-order formative construct, rendering PLS-SEM a suitable methodology for estimating parameters within hierarchically structured latent variable models involving reflective associations (Becker et al., 2012). It is pertinent to note that PLS-SEM does not impose the prerequisite for constructs to adhere to multivariate normality (Ringle et al., 2012).

In this section, firstly, the common method bias (CMB) was checked. Secondly, we evaluated measurement model for all the first-order reflective constructs. Thirdly, the estimate and assessment of the second-order formative construct (i.e., intrinsic motivations) was presented. Lastly, we tested the research model and hypothesis.

#### 5.4.1 Common method bias

We checked the CMB by two methods. Initially, a Harman’s single-factor analysis was executed using SPSS 23.0, as detailed by Podsakoff et al. (2003). Results show that the first factor only accounts for 32.152% of the total variance, which indicates the inexistence of CMB in this study.

Subsequently, we adopted a PLS-SEM approach derived from Liang et al. (2007) to further evaluate the presence of CMB. The findings revealed that he variance on average attributed to the substantive constructs was 72.158%, whereas that explained by the common method factor stood at 0.952%. This resulted in a ratio of approximately 75:1. Overall, it can be concluded that CMB is not a significant concern within our dataset.

#### 5.4.2 Measurement model

Internal consistency reliability of the constructs was assessed through the composite reliability (CR) and Cronbach’s α. As depicted in Table 6, all CR values surpass 0.8, and the Cronbach’s α values exceed 0.7, indicating robust construct reliability.

**Table 6:** Internal consistency reliability and convergent validity.

Construct	Items	α	CR	AVE	Factor loading
Self-development (SD)	SD1	0.864	0.918	0.788	0.908
	SD2				0.821
	SD3				0.930
Self-control (SC)	SC1	0.817	0.879	0.646	0.825
	SC2				0.822
	SC3				0.789
	SC4				0.777
Hedonic motivation (HM)	HM1	0.798	0.881	0.712	0.834
	HM2				0.835
	HM3				0.862
Financial reward (FR)	FR1	0.770	0.867	0.684	0.797
	FR2				0.837
	FR3				0.846
Social recognition (SR)	SR1	0.870	0.912	0.721	0.893
	SR2				0.820
	SR3				0.830
	SR4				0.850
Intention for continued use (CS)	CS1	0.838	0.902	0.755	0.943
	CS2				0.850
	CS3				0.810

Convergent validity was checked via factor loadings and average variance extracted (AVE). Table 6 show that the factor loadings exhibit a span from 0.777 to 0.943, and the AVE values for all constructs surpass the threshold of 0.6. These findings support the sound convergent validity.

The assessment of discriminant validity encompassed two approaches: the Fornell–Larcker criterion and the heterotrait–monotrait ratio of correlations (HTMT) criterion. As depicted in Table 7, the square root of AVE for each construct surpasses its corresponding squared correlation coefficient with other constructs. The highest recorded HTMT value stands at 0.632 (Table 8), a value below the 0.85 threshold. The upper bounds of the 97.5% confidence intervals consistently remain below 1, thus indicating the statistically significant disparity of all HTMT values from unity. Collectively, these findings provide compelling evidence supporting the robust discriminant validity.

**Table 7:** Correlation matrix and square root of AVEs.

Construct	1	2	3	4	5	6
1. Self-development	**0.888**					
2. Self-control	0.496	**0.804**				
3. Hedonic motivation	0.351	0.486	**0.844**			
4. Financial reward	0.180	0.154	0.071	**0.827**		
5. Social recognition	0.225	0.176	0.172	0.042	**0.849**	
6. Intention for continued use	0.525	0.536	0.452	0.366	0.495	**0.869**

Bold values represent the square root of AVE, and the others represent the inter-construct correlations.

**Table 8:** Heterotrait–monotrait ratio of correlations values and confidence intervals (2.5, 97.5%).

Construct	1	2	3	4	5	6
1. Self-development						
2. Self-control	0.594 (0.523, 0.658)					
3. Hedonic motivation	0.425 (0.344, 0.511)	0.600 (0.516, 0.675)				
4. Financial reward	0.218 (0.125, 0.321)	0.189 (0.098, 0.292)	0.090 (0.047, 0.216)			
5. Social recognition	0.257 (0.177, 0.336)	0.211 (0.117, 0.298)	0.207 (0.104, 0.298)	0.059 (0.039, 0.153)		
6. Intention for continued use	0.606 (0.531, 0.677)	0.632 (0.566, 0.698)	0.534 (0.449, 0.615)	0.441 (0.335, 0.529)	0.564 (0.486,0.632)	

#### 5.4.3 Second-order construct estimate and assessment

The estimation of a second-order formative construct can be achieved through two primary methodologies: the two-stage approach and the repeated indicators approach (Ringle et al., 2012). In this study, the construct of intrinsic motivations was estimated by the two-stage approach for two reasons. First, the repeated indicators approach is more suitable when the first-order constructs possess an equal number of indicators (Luo et al., 2010). Our three first-order dimensions of intrinsic motivations have different numbers of indicators. Second, according to Becker et al. (2012), the two-stage approach should be applied if the researcher focus lies solely on the higher-level estimations. While our main interest of Study 2 focuses on the effects of extrinsic motivations on intrinsic motivations and how they affect intention for continued use. Following Ringle et al. (2012), we first calculate the scores of the first-order constructs, and employ these scores as formative indicators for the second-order construct.

The assessment of measurement quality for the formative second-order construct necessitates an evaluation encompassing correlations among the first-order constructs, the weights assigned to the indicators, the statistical significance of these weights, and an examination for potential issues of multicollinearity (Becker et al., 2012). First, the correlations of three dimensions of intrinsic motivations are: 0.496 (self-development and self-control), 0.351 (self-development and hedonic motivation), and 0.486 (self-control and hedonic motivation). These results indicate intrinsic motivations should be a formative construct, as a reflective construct would have correlations higher than 0.8 among its indicators (Pavlou and El Sawy, 2006). Second, the weights of self-development, self-control and hedonic motivation, respectively, are 0.535, 0.420 and 0.292, and all of them are significant at 0.001. Third, the variance inflation factor (VIF) are used to check multicollinearity. Results show that all VIF values are lower than 3, indicating multi-collinearity is not a concern.

#### 5.4.4 Structural model

**Figure 3:**
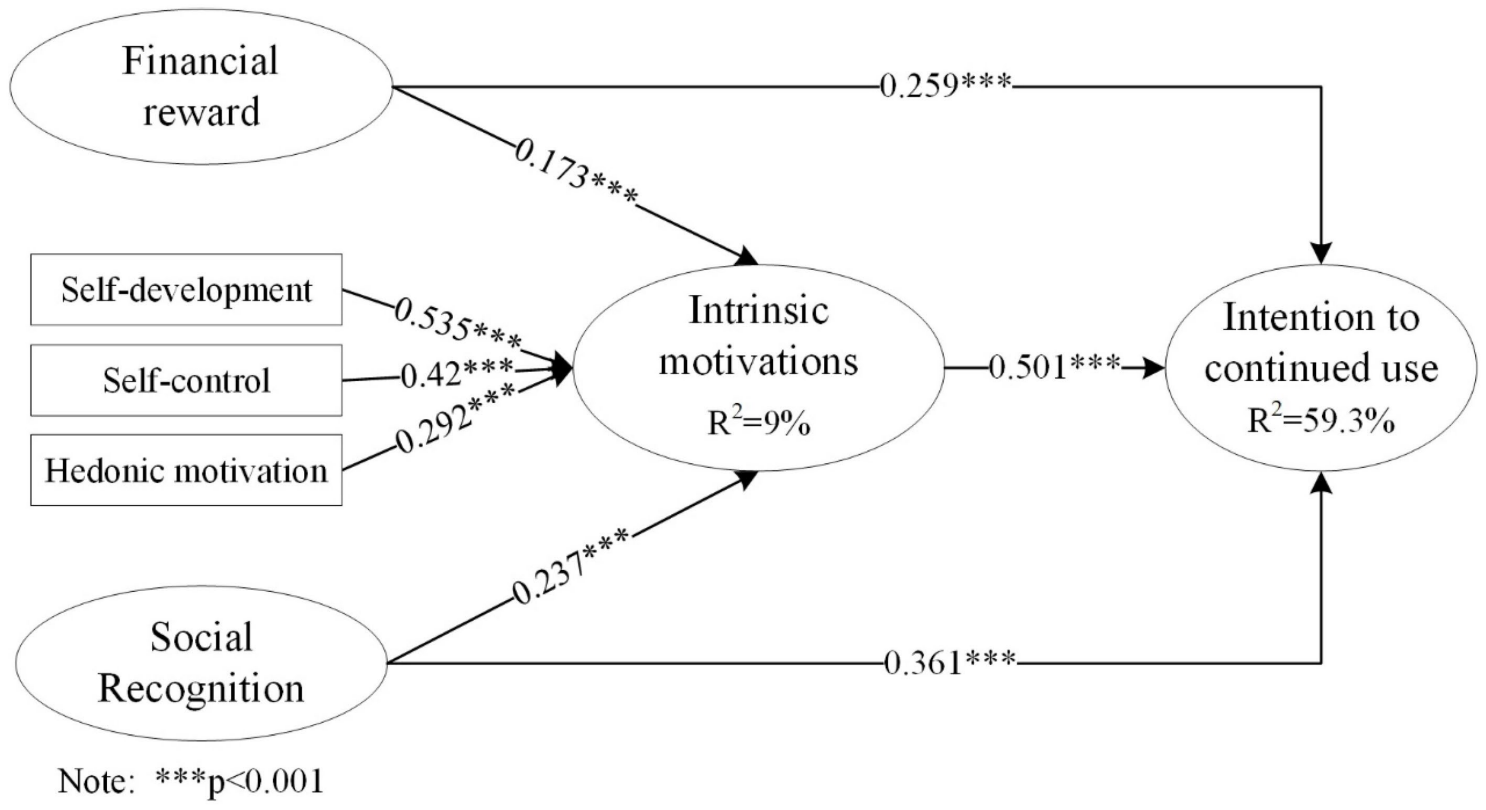
Results of structural model.

Figure 3 illustrates the examination of hypotheses test. Our research model demonstrates an explanatory capacity of 59.3% of the variance in the intention for continued use, and 9% of the variance in intrinsic motivations. Intrinsic motivations (β = 0.501, *p* < 0.001) exhibit a positive influence on intention for continued use, thus supporting H1. Correspondingly, financial reward (β = 0.259, *p* < 0.001) showcases a positive association with intention for continued use, substantiating H2. Furthermore, social recognition (β = 0.361, *p* < 0.001) demonstrates a positive correlation with intention for continued use, supporting to H3.

As for the motivation crowding effects, both financial reward (β = 0.173, *p* < 0.001) social recognition (β = 0.237, *p* < 0.001) are found to be positively associated with intrinsic motivations. Consequently, H4 and H5 are empirical supported.

We calculated the Cohen’s ƒ^2^ to measure the effect size of each path.^1^ Results indicate the Cohen’s ƒ^2^ values in our model ranges from 0.033 to 0.560 (small to large): the effect size of “intrinsic motivations—intention for continued use” (ƒ^2^ = 0.560) is large; the effect sizes of “financial reward—intention for continued use” (ƒ^2^ = 0.160) and “social recognition—intention for continued use” (ƒ^2^ = 0.302) are medium; the effect sizes of “financial reward—intrinsic motivations” (ƒ^2^ = 0.033) and “social recognition—intrinsic motivations” (ƒ^2^ = 0.062) are small. Table 9 summarizes the coefficient, *p*-values, Cohen’s ƒ^2^ of each path.

**Table 9:** The coefficients, *p*-values and Cohen’s *f*^2^ of each path.

Independent	Dependent	Coefficient	*p*-value	Cohen’s *f*^2^
Intrinsic motivations	Intention for continued use	0.501	0.000	0.560
Financial reward	Intrinsic motivations	0.173	0.000	0.033
	Intention for continued use	0.259	0.000	0.160
Social recognition	Intrinsic motivations	0.237	0.000	0.062
	Intention for continued use	0.361	0.000	0.302

Furthermore, to examine the mediating effects of intrinsic motivations, we adopted the methodology outlined by Vance et al. (2015). According to the results shown in Table 10, the effects of financial reward and social recognition on continued use intention are both partially mediated by intrinsic motivations. As for financial reward, the direct and indirect effects on the intention for continued use are 0.259 and 0.087, respectively; thus, the total effect is 0.346 (Table 10). As for social recognition, the direct and indirect effects on the intention for continued use are 0.361 and 0.119, respectively; thus, the total effect is 0.480 (Table 10). These results indicate that both financial reward and social recognition could increase intention for continued use via the mediation effects of intrinsic motivations, supporting the crowd-in effects of both financial reward and social recognition on intrinsic motivations.

**Table 10:** Bootstrapped confidence interval tests for mediation.

Variable	Mediation test (ab)			Full/partial mediation test (c’)			Type of mediation	Direct effect	Indirect effect	Total effect
	2.5% lower bound	97.5% upper bound	Zero included?	2.5% lower bound	97.5% upper bound	Zero included?				
Financial reward	0.043	0.132	No	0.204	0.313	No	Partial	0.259	0.087	0.346
Social recognition	0.081	0.159	No	0.306	0.415	No	Partial	0.361	0.119	0.480

## 6 Discussion

This current study tries to provide insights into the roles of user motivations in continued use intention to gamified fitness apps by addressing two research questions: “RQ1: How do intrinsic and extrinsic motivations could drive continued use, respectively?” and “RQ2: Further, do extrinsic motivations crowd-in or crowd-out intrinsic motivations?.” First, a qualitative investigation employing semi-structured interviews was executed to elucidate the underlying motivations for using gamified fitness apps, and these identified motivations were then treated as main factors in the research model in qualitative study. Second, a survey quantitatively confirmed the significant effects of both intrinsic and extrinsic motivations on continued use intention, as well as the motivation crowd-in effect, in gamified fitness apps context. Our key findings are discussed below.

Results of qualitative study show that gamified fitness apps use could be driven concurrently by three intrinsic motivations (self-development, self-control and hedonic motivation) and two extrinsic motivations (social recognition and financial reward). These results confirm to the literatures indicating that gamified services are motivated both intrinsically and extrinsically (Hamari and Koivisto, 2015; Jang et al., 2018; Wolf et al., 2020), and that gamification affordances can provide both intrinsic and extrinsic motivations (Mulcahy et al., 2020). That is, these results suggest that user’s motivations for using gamified fitness apps could be complicated and mixed. Through interaction with elements such as points, feedbacks and challenge, users can use gamified fitness apps to have a feeling for improvement of their well-being, controlling themselves, and fun or pleasure. These kinds of experiences can intrinsically motivate use of gamified fitness apps. Besides, users could also use gamified fitness apps to satisfy their extrinsic needs for financial reward and social recognition, through interacting with social-related elements and financial reward. These results can help understand what specific intrinsic and extrinsic motivations could arise in gamified fitness apps from a user-centric perspective.

Based on the results from qualitative study, a research model was proposed to address the two research questions by investigating the effects of both intrinsic and extrinsic motivations on continued use intention, and the motivations crowding effects. As for RQ1, empirical results show that both intrinsic and extrinsic motivations significantly drive continued use intention. Intrinsic motivations (β = 0.501, *p* < 0.001) are found to be the most important factor for continued use intention. Hence, the most important reason for users to continuously use gamified fitness apps is to satisfied their intrinsic needs for self-development, self-control and hedonic needs. Extrinsic motivations, including social recognition (β = 0.361, *p* < 0.001) and financial reward (β = 0.259, *p* < 0.001), are also found to positively affect continued use intention. Some users hope to gain positive social feedbacks such as “like” or attention of others when they complete the fitness challenge, while some users aims to get tangible rewards through using the apps. These results indicate that users could be extrinsically motivated to continuously use gamified fitness apps when receiving financial reward and positive social recognition.

As for RQ2, empirical results support the crowd-in effects in gamified fitness apps. Financial reward (β = 0.173, *p* < 0.001) is found to positively affect intrinsic motivations, suggesting that providing financial reward in the proper way can let user feel better experience related to intrinsic motivations including self-development, self-control or hedonic motivations. Besides, social recognition (β = 0.237, *p* < 0.001) is also found to positively affect intrinsic motivations. This finding is consistent with literatures that indicate social recognition might create the feelings of belonging and social relatedness which is an intrinsic need satisfaction (Shiau et al., 2018; van Roy and Zaman, 2019).

Results of mediation test indicate that intrinsic motivations could partially mediated the effects of financial reward and social recognition on continued use intention. These mediation effects indicate that these two extrinsic motivations could have direct and indirect effects on continued use intention, and the latter is via enhancing intrinsic motivations that in turn drive continued use intention, which further verify the crowd-in effects. That is, users’ who receive financial reward and social recognition from the services would be more intrinsically motivated to use the services. The total effects of intrinsic motivations, financial reward and social recognition on continued use intention are namely 0.501, 0.346, and 0.480. In general, although prior gamification studies mainly focus on the non-financial benefits of gamification (Jang et al., 2018), our results highlight the importance of extrinsic motivations in the gamified fitness apps context by confirming their direct effects on continued use intention and motivation crowd-in effects on intrinsic motivations. These findings could provide insights into the roles of user motivations by revealing how extrinsic motivations and intrinsic motivations work together in driving continued use intention in gamified fitness apps.

### 6.1 Theoretical implications

Our findings could contribute to the literature related to fitness apps, gamification and MCT in several ways. Our first contribution is that we investigated the motivation crowding effects in the gamified fitness apps contexts by testing the effects of extrinsic motivations on intrinsic motivations. This is important because gamified fitness apps can incorporate various game design elements to both intrinsically and extrinsically motivate users. From the perspective of MCT, extrinsic motivations possess the potential to either crowd-out or crowd-in intrinsic motivations in different identifiable conditions (Frey and Jegen, 2001). Literature called for more attention the motivation crowding effects in various IS contexts (von Krogh et al., 2012), especially in gamified systems (Liu et al., 2017). However, empirical evidence on the motivation crowding effects in the gamified fitness apps is rare. We confirm the crowd-in effects in the gamified fitness apps by finding that both financial reward and social recognition could positively affect intrinsic motivations, and further, intrinsic motivations could have mediating effects on these two extrinsic motivations. Examining the motivation crowding effects can enrich theorization of user motivations by providing context-specific insights into the interplay and potential conflicts between diverse forms of motivation (Wu, 2019). Fundamentally, this research contributes to the expansion of our understanding by accentuating the phenomena of motivation crowd-in within the realm of gamified fitness applications.

Second, this study advances knowledge on continued use of gamified fitness apps by revealing the roles of intrinsic motivations extrinsic motivations. Although literatures have found significant factors for intention for continued use of fitness apps from various theoretical perspectives such as TAM, UTAUT, and ECM (Cho et al., 2015; Li et al., 2019), it’s likely that additional factors that are left to explored. Especially, the roles of user motivations on continued use intention to gamified fitness apps need further study. Gamified fitness apps incorporate a variety of game design elements to enhance the systems with motivational affordances for game-like experiences. In addition to access to the basic fitness services, users could interact with PBL, social with others, challenge, make a team, gain reward and so on. User motivations have been confirmed as significant factors for fitness app use (James et al., 2019). Different from studies drawing from typical theory for IS use, we use a mix-method design to reveal the roles of two extrinsic motivations (i.e., financial reward and social recognition) and three intrinsic motivations (i.e., self-development, self-control and hedonic motivation) in driving intention for continued use gamified fitness apps. These findings can gain a more specific understanding of the reasons for continually using gamified fitness apps from a user-centric perspective. In addition, these findings can respond to the call by Koivisto and Hamari (2019) that “future gamification research should pay more effort into the pre-determinants of gamification success, and become less tightly focused on the affordances and outcomes of gamification.” Understanding the roles of specific user motivations on continued use intention can enhance our ability to foster the long-term success of gamified fitness apps.

Third, this study extended the MCT by highlighting the crowd-in effects of social recognition to the new research context, i.e., gamified fitness apps. Most studies on MCT have investigated the motivation crowding effect by focusing on the relationship between financial rewards and intrinsic motivations, which were conducted in the contexts of employee behavior (Dierynck et al., 2012; Torre et al., 2020), public service behavior (Corduneanu et al., 2020), prosocial behavior (Graafland, 2020). This study stands among the limited number of investigations on the crowding effects of social recognition on intrinsic motivations in the context of gamified fitness apps. This underscores the significance of comprehensively assessing both the immediate and mediated impacts of social recognition on usage of gamified fitness apps, as well as in analogous contexts of gamified services.

### 6.2 Practical implications

These findings provide some practical implications for the designers of gamified fitness apps. Literature has highlighted the challenge for long-term success of gamified fitness apps (Koivisto and Hamari, 2019). Thus, it’s crucial for designers to understand the underlying psychological mechanism for continued use of gamified fitness apps, which is important requirements of gamification success. In general, our findings indicate that user motivations, including intrinsic motivations and extrinsic motivations, can effectively explain intention for continued use of gamified fitness apps. Hence, it is recommended that apps designers pay close attention to user intrinsic and extrinsic motivations for using gamified fitness apps. Before simply adding a set of “standardized” game design elements to the apps, managers should consider carefully which specific elements to use to satisfy users’ various needs, which in turn enhance users’ different motivations.

On the one hand, given the crucial role of intrinsic motivations in continued use intention, gamified fitness apps should be designed to enhance users’ intrinsic motivations. According to the findings, a reasonable approach could be providing users with feelings of self-development, self-control and hedonic experiences in the with the apps, by incorporating various game design elements. For self-development, the gamified fitness apps should provide users with professional knowledge to help users improve their abilities and skills; and immediate feedback so that users can feel their progress when they make a progress. In terms of self-control, suitable goals should be set for different users to help them keep exercise for stick to a long-term exercise plan; also, users should be reminded and encouraged when they are detected not to exercise consistently. In terms of hedonic motivation, immersion-related gamification affordances such as character, story, music and even mini-games should be incorporated in the services.

On the other hand, considering the significant roles of extrinsic motivations on continued use intention as well as the crowd-in effects, gamified fitness apps should be designed to improve users’ extrinsic motivations. Our findings suggest that users could be extrinsically motivated to continually use by social recognition and financial reward. As for social recognition, the apps designer should guarantee that users can receive positive social feedbacks from others when they make a progress in gamified fitness apps. Social gamification affordance such as leaderboards, cooperation, competition and praise should be used to provide users with social recognition. As for financial reward, it’s suggested that modest financial reward should be provided to users for achieving exercise goals or in turn for the cumulative points.

### 6.3 Limitations and future research

We must acknowledge that this study has some limitations. First, this study highlights the motivation crowding effects in gamified fitness apps. However, this study does not investigate how specific game design elements could affect different motivations. We recognize the need for a more in-depth exploration of the logic chain of “game design elements—user motivations—continued use.” Specifically, future research could delve into the specific impacts of various game design elements (e.g., PBL, team, competition and challenges) on extrinsic motivations (e.g., perceptions of social recognition and financial rewards) or intrinsic motivations (e.g., self-development, self-control and hedonic motivation). By addressing these aspects in more detail, researchers can contribute to comprehensive examination of the motivation crowding effects in gamified fitness apps. Secondly, the model developed in this study, rooted in qualitative findings and the framework of Motivation Crowding Theory, does not account for individual variations such as age, gender, and technological innovativeness. To bolster the robustness of the model, future investigations could integrate these potential covariates. Third, our research sample only included users of certain gamified exercises in China. Given the potential impact of cultural disparities, we advocate for future studies to apply the model across more diverse samples to enhance its generalizability. In addition, all research constructs were measured by self-report questionnaires, which might result in the problem of response bias. Future research could adopt experimental methodologies to procure objective data to test the model.

## Data availability statement

The raw data supporting the conclusions of this article will be made available by the authors, without undue reservation.

## Ethics statement

Ethical approval was not required for the studies involving humans in accordance with the local legislation and institutional requirements. Written informed consent for participation was not required from the participants or the participants’ legal guardians/next of kin in accordance with the national legislation and institutional requirements.

## Author contributions

JH: Writing – original draft. JC: Writing – review & editing. LZ: Writing – review & editing.

## Footnotes

^1^Cohen’s ƒ^2^ 0.02, 0.15, and 0.35 are considered as small, medium, and large effect sizes.
